# Avenues for medical student research at an institution without a home residency program

**DOI:** 10.3389/fmed.2026.1944698

**Published:** 2026-09-16

**Authors:** Daniel C. Lee, Baotram Nguyen, Brian Grodecki, Meghan K. Berkenstock

**Affiliations:** 1 Department of Medicine, Drexel University College of Medicine, Philadelphia, PA, United States; 2 Division of Ocular Immunology, Wilmer Eye Institute, Johns Hopkins School of Medicine, Baltimore, MD, United States

**Keywords:** medical student, ophthalmology, research, research arms race, residency, specialty, subspecialty

## Abstract

**Introduction:**

The goal of this study is to explore the strategies and challenges in obtaining research experiences among medical students pursuing ophthalmology, other surgical subspecialties (OSS), and non-surgical specialties (NSS) at an institution without a home residency for these specialties.

**Methods:**

A 20-question confidential survey was distributed to Drexel University College of Medicine medical students spanning 1st year to 4th year and descriptive analyses were utilized to convey our results.

**Results:**

Of 93 respondents, there were 22 (33.3%) in ophthalmology, 32 (48.5%) in OSS, and 12 (18.2%) in NSS, while 27 students had incomplete responses or did not specify a specialty preference. Sixty-one respondents (92.4%) were from the Philadelphia campus. The mean confidence in having sufficient research experience to be a competitive applicant was comparable across groups (ophthalmology: 2.76; OSS: 2.67; NSS: 3.50; *p* = 0.219). Cold emailing was the most common method of securing initial research (ophthalmology: 52%; OSS: 38%; NSS: 55%), while DUCOM faculty, pathway directors, or alumni connections were underutilized. The most frequently cited challenges were a lack of mentors and difficulty identifying opportunities. Ophthalmology and OSS students placed higher importance on research compared to NSS students. Across all specialties, research acquisition was largely self-directed, with limited use of faculty and access to alumni. Despite similar levels of research participation, surgical specialties, including ophthalmology, reported greater perceived barriers and desire for support.

**Discussion:**

At institutions without specialty-specific home residency programs, early peer mentorship, structured research orientation, and alumni engagement may improve equity in research access and preparation for competitive specialties.

## Introduction

Securing a position in today's residency landscape increasingly hinges on demonstrable research output. Across every specialty, matched allopathic medical school seniors in the United States now report two-to-three-fold more abstracts, presentations, and publications than a decade ago (1, 2). In highly competitive fields, such as dermatology and neurosurgery, the average routinely exceeds a combination of 20 research manuscripts and presentations per applicant. National Resident Matching Program (NRMP) data confirm that greater research experience correlates with higher match success, even after accounting for board scores and other metrics. With Step 1 score now reporting as pass or fail, program directors have placed a heavier reliance on research productivity to differentiate candidates, intensifying what has been referred to as a “research arms race.” (3)

Yet access to meaningful research opportunities is far from uniform. Students at institutions lacking home residency programs (HRPs) in competitive surgical subspecialties frequently describe limited exposure to faculty mentors, fewer in-house projects, and the need to depend upon less reliable and more time-consuming networking methods, such as cold emailing. For example, in plastic surgery, applicants without an HRP felt disadvantaged in finding mentors and learning about research. Only 17% of residents at top-quartile programs came from HRP-absent schools (3, 4). Similar concerns have been raised in dermatology and other fields, where alumni connections and structured mentorship have been proposed as partial solutions (4, 5). Understanding how students navigate these barriers is essential for creating equitable support systems for medical students without an HRP in their specialty of interest.

We surveyed medical students at Drexel University College of Medicine (DUCOM), a Philadelphia and West Reading-based medical school without home programs in ophthalmology, otolaryngology, urology, plastic surgery, neurosurgery, orthopedics, and other surgical subspecialties, to identify the strategies students use to secure research, their confidence in those experiences, and the commonly encountered obstacles. By comparing responses across ophthalmology, other surgical subspecialties, and non-surgical fields, we aim to highlight actionable initiatives that could broaden research access and improve competitiveness for students regardless of institutional resources.

## Methods

An anonymous 20-question survey (Table 1) was administered through Qualtrics and was accessible only to users logged in with a DUCOM e-mail address to ensure the survey was limited to active medical students. Qualtrics internally records email addresses, limiting students to one submission while ensuring anonymity. The survey link was distributed once to all medical students (years one through four; 1,240 students) via a class-wide e-mail and shared in each class's (class of 2025, 2026, 2027, 2028) GroupMe channels and relevant specialty interest group messaging boards. GroupMe is a free group messaging application that facilitates communication among groups, often used by student organizations in medical school. The survey remained open from January 24, 2025, through February 9, 2025. Participation was voluntary; students were allowed to discontinue at any time, and no incentives were offered.

**Table 1:** Survey questions. List of survey questions that were distributed to medical students with response rate (*n* = 66 total responses).

Question category	Question (*N*)	Answer choices (*n*, %)	Total Response (*N*, %)
Demographics	What campus do you attend? (66)	Philadelphia (61, 92.4%) West Reading (5, 7.6)	66, 100%
	What is your current year of medical school? (66)	M1 (22, 33.3%) M2 (11, 16.7%) M3 (17, 25.8%) M4 (16, 24.2%)	66, 100%
	What is your current educational background outside of pursuing an MD? (60)	BS (42, 70%) BA (18, 30%)	60, 90.9%
Single choice	Are you currently involved in research or have you been involved in research since starting medical school? (66)	Yes (56, 84.8%) No (10, 15.2%)	66, 100%
	Did you complete any research experiences before medical school? (66)	Yes (57, 86.4%) No (9, 13.6%)	66, 100%
	Have you continued with this research since starting medical school? (55)	Yes (24, 43.6%) No (31, 56.5%)	55, 83.3%
	When did you first start doing research in medical school (or when are you planning on doing research, if at all)? (64)	1st year August-December (20, 31.3%) 1st year January-May (23, 35.9%) 1st year summer (18, 28.1%) 2nd year (0, 0%) 3rd year (3, 4.7%) 4th year (0, 0%)	64, 96.9%
	Which of the following sources was most valuable in locating research in medical school? (65)	Faculty-led zoom (13, 20%) Mentors (26, 40%) Medical student panel (15, 23.1%) Classmates (7, 10.8%) Self-directed (1, 1.5%) Other (3, 4.6%)	65, 98.5%
	How did you obtain your first research experience? (61)	Previous mentors (4, 6.6%) Cold-emailing (28, 45.9%) Pathway/faculty (11, 18%) Drexel opportunity (4, 6.6%) Other (14, 23.0%)	61, 92.4%
	Were you able to find any research through a family connection? (60)	Yes (6, 10%) No (54, 90%)	60, 90.9%
Multiple choice (select all that apply)	If you participated in research, what type of research did you do or have you done? (62)	Clinical (54, 87.1%) Translational (27, 43.5%) Basic science (16, 25.8%)	62, 93.9%
	Which of the following were part of the application process for obtaining your first research position? (45)	Formal application (14, 31.1%) CV (38, 84.4%) Cover letter (8, 17.8%) Interview (22, 48.9%)	45, 68.2%
	In what years did you participate in research? (60)	Prior to medical school (45, 75.0%) M1 (36, 60.0%) M2 (29, 48.3%) M3 (12, 20.0%) M4 (2, 3.3%) Does not apply (45, 75.0%)	60, 90.9%
	What was/were your greatest challenge(s) when reaching out to PIs or applying for research opportunities? (26)	Receiving a response (17, 65.4%) Finding a PI taking students (9, 34.6%) Location (14, 53.8%) Paid position (18, 69.2%) In area/field of interest (2, 7.7%) Knowing where to start (3, 11.5%)	26, 39.4%
	What was/were your greatest challenge(s) with the research process? (60)	Learning about opportunities (19, 31.7%) Understanding requirements (13, 21.7%) Finding mentors (48, 78.3%) Finding research I was interested in (29, 48.3%) Other (5, 8.3%)	60, 90.9%
Quantitative	How much importance do you place on research involvement during your medical school career? (60) (mean, median)	1–10, 10 being extremely important (8.2, 9)	60, 90.9%
	Rate your level of confidence that you will have enough research experience to be a competitive applicant in your specialty of interest. (54) (mean, median)	1–5, 5 being extremely confident (2.85, 3)	54, 81.8%
Free Response (optional)	What are your specialties or areas of interest? (66)	—	66, 100%
	Comment on your research experience as a DUCOM student. (27)	—	27, 40.9%
	Describe what type of support DUCOM can provide students interested in research to improve their experience. (27)	—	27, 40.9%

Inclusion criteria were enrollment as a first-year through fourth-year medical student (M1–M4) at DUCOM during the study period and completion of the survey. Participants who did not report a specialty of choice or answer non-demographic questions were excluded due to the inability to categorize responses into the groups listed below. As long as the participant completed the demographic section and responded to at least one other survey question, they were included for analysis. Respondents self-identified an intended specialty, which was collapsed into three categories for analysis: ophthalmology, other surgical subspecialties excluding ophthalmology (OSS) [which included otolaryngology, urology, plastic surgery, neurosurgery, orthopedics, cardiothoracic surgery, general surgery, and obstetrics-gynecology], and non-surgical specialties (NSS) [which included internal medicine and its subspecialties, interventional radiology, anesthesia, neurology, dermatology, emergency medicine, pediatrics, family medicine, medicine-pediatrics, and psychiatry]. Each respondent was allowed to enter more than one field.

Ophthalmology was selected to be seperate from other surgical subspecialties because it utilizes a seperate residency portal, and our institution's affiliate hospitals do not have any respective ophthalmology residencies. When ophthalmology was one of several specialties listed, the respondent was classified under ophthalmology. Respondents who indicated multiple specialty interests were classified based on the most specialized or most surgical field listed due to the likelihood of truly reflecting their approach to research involvement. For example, if a student did not list ophthalmology and listed general surgery and internal medicine, they were grouped into OSS. Key outcome variables were (a) the method by which students secured their first research experience, (b) the greatest perceived challenges when seeking research, and (c) confidence (1 = not at all, 5 = extremely) that their research portfolio would be competitive for application to residency in their field of interest. Explanatory variables included training year, prior degrees, and the presence of family connections in medicine.

Survey data were first exported from Qualtrics, in which responses were automatically assigned a numeric code. Data analysis was conducted with SPSS (Version 30; IBM Corp., Armonk, NY). Analysis for statistical differences in numeric rating survey questions was conducted using a non-parametric one-way analysis of variance (ANOVA), with *post hoc* Tukey's multiple comparisons test used to examine the three specialty categories for statistically significant differences. Analysis for statistical differences in multiple choice survey questions was conducted using Fisher's exact test, with *post hoc* Bonferroni corrected Chi-square test used to examine the three specialty categories for statistically significant differences. Statistical significance was determined by *p*-value ≤ 0.05. A descriptive analysis was performed on free responses to identify common themes. Tables were designed with Excel for Windows (Version 2409 Build 16.0.18025.20030; Microsoft Corp, Redmond, Wash.).

The Drexel University College of Medicine Institutional Review Board reviewed and approved the protocol (IRB#2410010850). They determined the study to be exempt as de-identified anonymized survey data were analyzed, and a waiver of written informed consent was granted. Participation was voluntary, students were allowed to discontinue at any point, and informed consent to participate was obtained from all participants in the study during the first page of the survey. The study was conducted in accordance with the ethical principles of the Declaration of Helsinki.

## Results

Ninety-three total participants were recorded to have attempted the survey; 66 of them answered at least one question in addition to their demographics, while 27 excluded entries did not indicate a specialty of choice or did not complete the demographics questions. Of the 66 respondents (5.3% response rate), 61 participants (92.4%) were from the Philadelphia campus, while 5 participants (7.6%) attended the West Reading campus. Twenty-two participants (33.3%) were M1s, 11 (16.7%) were M2s, 17 (25.8%) were M3s, and 16 (24.2%) were M4s. Twenty-two students (33.3%) indicated interest in ophthalmology, 32 (48.5%) in OSS, and 12 (18.2%) in NSS. There were no significant differences in demographics amongst the specialty categories (Table 2).

**Table 2:** Student demographics. Total of 66 medical students completed the entire survey. Anonymous demographic distribution is listed in the table.

		Specialty							
		Ophthalmology		OSS		NSS		Total	
Characteristics		*n*	% within specialty	*n*	% within specialty	*n*	% within specialty	*n*	%
Campus	Philadelphia	19	86.4%	30	93.8%	12	100.0%	61	92.4%
	Reading	3	13.6%	2	6.3%	0	0.0%	5	7.6%
	Total	22	100.0%	32	100.0%	12	100.0%	66	100.0%
Year	M1	12	54.5%	7	21.9%	3	25.0%	22	33.3%
	M2	2	9.1%	7	21.9%	2	16.7%	11	16.7%
	M3	3	13.6%	10	31.3%	4	33.0%	17	25.8%
	M4	5	22.7%	8	25.0%	3	25.0%	16	24.2%
	Total	22	100.0%	32	100.0%	12	100.0%	66	100.0%
Education	BS	17	85.0%	17	58.6%	8	72.7%	42	70.0%
	BA	3	15.0%	12	41.1%	3	27.3%	18	30.0%
	Total	20	100.0%	29	100.0%	11	100.0%	60	100.0%

^a^ OSS: other surgical sub-specialties; NSS: non-surgical sub-specialties

The mean confidence in having sufficient research experience to be a competitive applicant was 2.76 out of 5 in ophthalmology students (*n* = 17, 31.5%), similar to the OSS at 2.67 (*n* = 27, 50.0%), and NSS at 3.5 (*n* = 10, 18.5%, *p* = 0.219). Median confidence across all subgroups was rated at 3. Mean student rating for importance of research during their medical school career significantly differed from surgical to non-surgical specialties: 8.65 in ophthalmology (*n* = 20, 33.3%), 8.4 in OSS (*n* = 29, 48.3%), and 6.9 in NSS (*n* = 11, 18.3%, *p* = 0.003), with 10 reflecting a high value placed on the need for research. The median ratings of research importance for ophthalmology, OSS, and NSS subgroups were 9, 9, and 8, respectively.

There was a significant difference across the specialty categories on research involvement prior to medical school, with 95.5% of ophthalmology students (*n* = 21), 75.0% of OSS students (*n* = 24), and 100% (*n* = 12) of NSS students reporting completing research experiences prior to medical school (*p* = 0.05). However, once students entered medical school, there did not appear to be a statistically significant difference in research participation, methods of finding research, or use of familial connections.

Cold emailing was the most common method of securing initial research (ophthalmology 52%, *n* = 11 OSS 38%, *n* = 11; NSS 55%, *n* = 6), whereas DUCOM faculty, pathway directors, or alumni connections were less frequently utilized (ophthalmology 28.6%, *n* = 6; OSS 13.8%, *n* = 4; NSS 8.2%, *n* = 1). Students across all specialties identified finding mentors and locating opportunities as key challenges when searching for projects. The most valuable resources were noted to be mentorship (ophthalmology 36.4%, *n* = 8; OSS 48.4%, *n* = 15; NSS 25.0%, *n* = 3), with students emphasizing the usefulness of faculty-led zoom meetings (ophthalmology 27.3%, *n* = 6; OSS 12.9%, *n* = 4; NSS 25.0%, *n* = 3), and medical student panels (ophthalmology 13.6%, *n* = 3; OSS 29.0%, *n* = 9; NSS 25.0%, *n* = 3).

The free response questions surveyed the type of support that would be most helpful to DUCOM students regarding research access and general comments about the student research experience. The most common themes included:

1. Lack of DUCOM research mentors in students' specific specialties of interest, resulting in requests for specialty-specific alumni connections and mentorship (*n* = 8).
2. Lack of accessible research, despite geographic location in an area with multiple other medical institutions (*n* = 4).
3. More alumni and/or faculty connections in specific specialties (*n* = 10).

Additional details of free responses can be referenced in supplementary material

## Discussion

This study examined how medical students at a United States medical school without HRPs in ophthalmology and other surgical subspecialties acquire research experiences, and whether their strategies and perceived barriers differ from those of their peers pursuing non-surgical fields. Notably, we found that across all specialty interests, the process of obtaining research was largely self-directed. Students interested in ophthalmology or other surgical subspecialties were more likely to rely on strategies such as cold emailing and obtaining research through DUCOM pathway directors. Pathway directors at Drexel are practicing physicians who mentor medical students interested in pursuing their specialty and who connect them with research or other opportunities. However, ophthalmology and OSS students did not express higher confidence in the sufficiency of their research portfolios compared to NSS, despite often taking more proactive steps. This suggests that although they may invest more time and effort into research, students pursuing surgical fields may still perceive limited institutional support and guidance. This was most evident in fields without an established departmental presence.

These findings also contribute to the larger conversation about evaluating research during the electronic residency application service (ERAS) process. Beginning in the 2026 residency cycle, the Association of American Medical Colleges has transitioned the previous ERAS “publication” section to a broader “Scholarly Work” section to “reflect a broader and more inclusive view of scholarly contributions” and “to help applicants present their work more clearly.” (3, 6, 7) While this reform is a meaningful step towards emphasizing quality over quantity over research, our data may suggest that it will not fully resolve the underlying access inequity. At well-resourced institutions, students will likely have ample opportunities for multiple mentored projects, whereas students without home programs will continue to build their portfolios through inefficient, self-directed outreach. For students at either institution, the quality of work is likely tied to their mentors' aims and goals, and these research-focused clinicians are typically affiliated with larger academic institutions. On the contrary, students from any background should be prepared and intentional when seeking research collaborations by learning of attendings' research interests or presenting a thoughtful research idea after conducting a literature review. In other words, while there may be an element of structural inequity with who has access to do meaningful research, self-motivation and preparation can be also foundational for quality research experiences.

Mentorship and connections within a department are vital, especially as research publication and activity trends continue to increase. Common themes included uncertainty of how to reliably find research experiences and a lack of both institutional and peer support. This pattern may reframe what is often characterized as applicant overproduction; students without home programs may not be padding applications with low-quality work but rather struggling to access mentored research at all. Such narratives parallel a multi-institution survey of plastic-surgery applicants without an HRP, in which 66% felt disadvantaged and 97% of those who ultimately matched cited an externally secured mentor as critical to success (4).

Three recurring avenues for improvement arose from the survey. First, near-peer guidance mattered as students who obtained early introductions from upper-class colleagues reported smoother entry into projects. Second, respondents repeatedly called for a searchable alumni database. A “sister-institution” pairing piloted in plastic surgery has begun matching students from schools without HRPs to mentors at partnering centers (4). Third, several students asked for structured orientation or mentorship sessions during the M1 year—an approach that contributed to successful matching in general surgery applicants (8). Additional resources include national specialty societies spearheading mentorship programs to increase mentorship access, such as the VISION Mentoring Program in ophthalmology or the FUTURE in Urology Program (9, 10).

The culture of mentorship also differs between surgical and NSS specialties. Students aiming for surgical careers often start seeking mentors early for shadowing experiences and to obtain research projects. Previous studies suggest that having a surgical mentor and participating in surgical research during the preclinical years increased a students' interest in a surgical career 3–12-fold more than peers without surgical mentorship (11). In competitive fields, like dermatology, having a connection to a residency program can be a decisive advantage. One analysis found that nearly one-third of successful dermatology applicants matched at an institution where they had done research with a mentor, and a similar proportion matched at their HRP with a mentor's support (12). Students without an HRP must recreate these connections from scratch. Collectively, these insights support building more systematic pipelines within one's own institution, such as connecting current medical students to prior alumni in these fields.

The urgency of such support is underscored by broader trends. NRMP reports show a two-to-three-fold rise in research items per matched applicant over the past decade, with the steepest increases in competitive surgical fields (1, 2, 13). Commentaries warn that this research culture amplifies institutional inequities, such that students at well-resourced programs may more readily accumulate publications. Conversely, medical students with similar work ethics at smaller programs may invest a significant amount of time in outreach and often in unfunded research experiences (3). Our data support the need for a more formal mentorship infrastructure.

Ophthalmology offers a case-in-point of the challenges in research and mentorship for a surgical subspecialty. It is a highly competitive field that operates via an independent match system (the San Francisco Matching Program, also known as SF Match) with an earlier timeline, leaving students less room to delay preparation. According to the Association of University Professors of Ophthalmology (AUPO) 2025 Webinar, a statistical difference in total publications was reported between students with and without a home program (total: 3.93 and 2.27, respectively) (14). When comparing students' involvement in research without Alpha Omega Alpha (AOA) or Gold Humanism Honor Society (GHHS) membership, 61% of students (*n* = 309/508) matched if they had research compared to a 41% (*n* = 28/68) without research (15). This suggests that research productivity or publications may positively impact an applicant's likelihood of matching. The number of hours dedicated to ophthalmology courses in Association of American Medical College approved schools has also been decreasing over the last 20 years (16). Consequently, students interested in ophthalmology frequently have minimal exposure and must seek opportunities for shadowing or other forms of clinical exposure to the specialty. Surveys have found that medical students perceive the overall competitiveness of ophthalmology as the top barrier to pursuing the field (17). This perception is compounded by limited exposure and lack of structured mentorship, particularly at institutions without an HRP, leaving students uncertain about how to begin building a competitive application. The SF match application even includes a supplemental question, asking the applicant “describe an important mentor and how that person has been helpful.” (14) In short, early mentorship and exposure to ophthalmology are necessary to develop an interest in the field.

Strengths of the present study include capturing perspectives across all training years and combining quantitative data with narrative content. Limitations include the inability to link responses to eventual match outcomes, a small sample size, and the single-institution design which may limit generalizability. With the survey design and generally low response rate, there is risk of sampling, selection, and nonresponse bias; those who decide to complete the survey may have stronger views on research opportunities rather than those who do not. As a result, it may not present as a full representation of the views at our institution. Because participants could list multiple intended specialties, we categorized each respondent by the most specialized surgical field, which introduces misclassification bias. The study was organized as such because competitive surgical subspecialties typically require more research compared to other non-surgical fields, possibly contributing to an altered perception and relationship to research in these students.

Future work should compile and analyze similar data points from recently matched students, compare institutions with and without HRPs, track the impact of structured alumni or society-based mentorship on research productivity and match success, and evaluate alternative early mentorship platforms. Additionally, future studies should encourage data gathering from both allopathic and osteopathic institutions to broaden applicability. Prospective studies linking early mentorship interventions to downstream outcomes, such as first-author publications or successful residency placement, would clarify where limited educational resources provide the greatest benefit.

## Conclusions

In summary, students at an institution without ophthalmology and other surgical HRPs acquire research largely through self-initiated outreach, with less systematic use of faculty or alumni networks. These findings, coupled with national data on escalating research expectations, suggest that medical school-initiated mentorship and alumni-engagement strategies may improve access to research at institutions with no specialty specific home residency programs.

## Data Availability

The raw data supporting the conclusions of this article will be made available by the authors, without undue reservation.
