# Supplementary material for: Avenues for medical student research at an institution without a home residency program
Source: Front Med (Lausanne). 2026 Sep 16;13:1944698. doi: 10.3389/fmed.2026.1944698 (PMC13623724; doi:10.3389/fmed.2026.1944698)
Supplement: Supplementary file 1 [file Table1.docx]

|  |  | Ophthalmology | | OSS | | NSS | | Total | |
| --- | --- | --- | --- | --- | --- | --- | --- | --- | --- |
|  |  | n | % within specialty | n | % within specialty | n | % within specialty | n | % |
| Campus | Philadelphia | 19 | 86.4% | 30 | 93.8% | 12 | 100.0% | 61 | 92.4% |
|  | Reading | 3 | 13.6% | 2 | 6.3% | 0 | 0.0% | 5 | 7.6% |
|  | Total | 22 | 100.0% | 32 | 100.0% | 12 | 100.0% | 66 | 100.0% |
| Year | M1 | 12 | 54.5% | 7 | 21.9% | 3 | 25.0% | 22 | 33.3% |
|  | M2 | 2 | 9.1% | 7 | 21.9% | 2 | 16.7% | 11 | 16.7% |
|  | M3 | 3 | 13.6% | 10 | 31.3% | 4 | 33.0% | 17 | 25.8% |
|  | M4 | 5 | 22.7% | 8 | 25.0% | 3 | 25.0% | 16 | 24.2% |
|  | Total | 22 | 100.0% | 32 | 100.0% | 12 | 100.0% | 66 | 100.0% |
| Education | BS | 17 | 85.0% | 17 | 58.6% | 8 | 72.7% | 42 | 70.0% |
|  | BA | 3 | 15.0% | 12 | 41.1% | 3 | 27.3% | 18 | 30.0% |
|  | Total | 20 | 100.0% | 29 | 100.0% | 11 | 100.0% | 60 | 100.0% |

**Supplemental Index**

**Supplemental Table 1. Demographics Distribution** OSS: Other surgical Subspecialty; NSS: Non-surgical subspecialty; BS: Bachelor of Science; BA: Bachelor of Arts.

|  |  | Ophthalmology | | Other surgical | | Non-surgical | | Total | |
| --- | --- | --- | --- | --- | --- | --- | --- | --- | --- |
|  |  | n | % | n | % | n | % | n | % |
| Are you currently involved in research or have you been involved in research since starting medical school? | Yes | 18 | 81.8% | 28 | 87.5% | 10 | 83.3% | 56 | 84.8% |
|  | No | 4 | 18.2% | 4 | 12.5% | 2 | 16.7% | 10 | 15.2% |
|  | Total | 22 | 100.0% | 32 | 100.0% | 12 | 100.0% | 66 | 100.0% |
| Did you complete any research experiences before medical school? | Yes | 21 | 95.5% | 24 | 75.0% | 12 | 100.0% | 57 | 86.4% |
|  | No | 1 | 4.5% | 8 | 25.0% | 0 | 0.0% | 9 | 13.6% |
|  | Total | 22 | 100.0% | 32 | 100.0% | 12 | 100.0% | 66 | 100.0% |
| Have you continued with this research since starting medical school? | Yes | 9 | 42.9% | 8 | 36.4% | 7 | 58.3% | 24 | 43.6% |
|  | No | 12 | 57.1% | 14 | 63.6% | 5 | 41.7% | 31 | 56.5% |
|  | Total | 21 | 100.0% | 22 | 100.0% | 12 | 100.0% | 55 | 100.0% |
| When did you first start doing research in medical school (or when are you planning on doing research, if at all)? | 1st year August-December | 8 | 36.4% | 9 | 30.0% | 3 | 25.0% | 20 | 31.3% |
|  | 1st year January-May | 6 | 27.3% | 12 | 40.0% | 5 | 41.7% | 23 | 35.9% |
|  | 1st year summer | 7 | 31.8% | 7 | 23.3% | 4 | 33.3% | 18 | 28.1% |
|  | 3rd year | 1 | 4.5% | 2 | 6.7% | 0 | 0.0% | 3 | 4.7% |
|  | Total | 22 | 100.0% | 30 | 100.0% | 12 | 100.0% | 64 | 100.0% |
| Which of the following sources was most valuable in locating research in medical school? | Faculty lead zoom | 6 | 27.3% | 4 | 12.9% | 3 | 25.0% | 13 | 20.0% |
|  | Mentors | 8 | 36.4% | 15 | 48.4% | 3 | 25.0% | 26 | 40.0% |
|  | Medical student panel | 3 | 13.6% | 9 | 29.0% | 3 | 25.0% | 15 | 23.1% |
|  | Classmates | 4 | 18.2% | 1 | 3.2% | 2 | 16.7% | 7 | 10.8% |
|  | Self-directed | 0 | 0.0% | 1 | 3.2% | 0 | 0.0% | 1 | 1.5% |
|  | Other | 1 | 4.5% | 1 | 3.2% | 1 | 8.3% | 3 | 4.6% |
|  | Total | 22 | 100.0% | 31 | 100.0% | 12 | 100.0% | 65 | 100.0% |
| How did you obtain your first research experience? | Previous mentors | 0 | 0.0% | 2 | 6.9% | 2 | 18.2% | 4 | 6.6% |
|  | Cold-emailing | 11 | 52.4% | 11 | 37.9% | 6 | 54.5% | 28 | 45.9% |
|  | Pathway/faculty | 6 | 28.6% | 4 | 13.8% | 1 | 9.1% | 11 | 18.0% |
|  | Drexel opportunity | 1 | 4.8% | 3 | 10.3% | 0 | 0.0% | 4 | 6.6% |
|  | Other | 3 | 14.3% | 9 | 31.0% | 2 | 18.2% | 14 | 23.0% |
|  | Total | 21 | 100.0% | 29 | 100.0% | 11 | 100.0% | 61 | 100.0% |
| Were you able to find any research through a family connection? | No | 3 | 15.0% | 3 | 10.3% | 0 | 0.0% | 6 | 10.0% |
|  | Yes | 17 | 85.0% | 26 | 89.7% | 11 | 100.0% | 54 | 90.0% |
|  | Total | 20 | 100.0% | 29 | 100.0% | 11 | 100.0% | 60 | 100.0% |

**Supplemental Table 2. Single-choice Questions and Response Distribution.**

|  | Ophthalmology | | Other surgical | | Non-surgical | | Total | |
| --- | --- | --- | --- | --- | --- | --- | --- | --- |
|  | n | Mean | n | Mean | n | Mean | n | Mean |
| How much importance do you place on research involvement during your medical school career? | 20 | 8.65 | 29 | 8.3793 | 11 | 6.9091 | 60 | 8.2 |
| Rate your level of confidence that you will have enough research experience to be a competitive applicant in your specialty of interest (5 being extremely confident) | 17 | 2.7647 | 27 | 2.6667 | 10 | 3.5 | 54 | 2.8519 |

**Supplemental Table 3. Numeric Questions and Response Distribution.**

**Supplemental Table 4: Multiple Answer Choices and Response Distributions.**

Multiple Choice Survey Questions (Fill in text)

**Which of the following sources was most valuable in locating research in medical school? - Other (please explain)**

Summary: interest groups (2), prior research mentors (2)

- Interest group meetings
- Club/Interest Group-organized Research Projects and Case Reports
- Combination of mentors from previous research experiences and self-directed searches
- Research from undergrad- outside of DUCOM because no one even answered me

**How did you obtain your first research experience? - Other (please explain)**

Summary: classmates/upperclassmen (7), haven’t obtained research yet (3), prior educational institution (2), summer program (1), mentorship zoom (1), Drexel research list (1)

- talking to other medical students
- Through a classmate
- Student mentor (upper classmen)
- fellow classmate connected me with drexel faculty
- learning about Rothman Spine research from older students
- Attending research meeting with PI per the recommendation of an upperclassmen
- Upperclassman friend was looking for someone to continue project; met this friend through an identity-based club
- Haven't gotten an opportunity yet
- Did not obtain a research experience yet
- Still in the process of finding research
- referred from post-bac prior to drexel
- Undergrad because no one answered
- Summer program
- Connecting with AGH mentorship zoom
- drexel research list

**What was/were your greatest challenge(s) with the research process? - Other (please explain) - Text**

Summary: time management balancing research with schoolwork (3), access to quicker projects like case reports (1), finding mentor (1), statistic support for projects (1)

- Finding research that will be productive (in terms of pubs, presentations) given the realistic amount of time I have to contribute. Specifically, I wish I had reliable access to case reports in addition to the more detailed and time-intensive research I am doing now. I think not having an affiliated hospital really hurt our ability to have consistent case reports, I'm hoping that I can snag a few as an M3 on rotations.
- Balancing time between required curriculum and research
- I wanted to find a lab that allowed me a balance between the work of medical school while also being able to contribute as much as possible to projects.
- finding a mentor who was going to help me be productive while also exploring my interests
- Finding support for research projects, ie. statistician

Free Response Summary and Raw Responses

(1) **(Optional) Comment on your research experience as a DUCOM student.**

Summary: Lack of DUCOM research mentors in specific field of interest (8), geographic proximity to other medical institutions is a plus, but research is not as accessible to outside students (4), took a research year to bolster residency application (3), difficulty finding research that is productive and results in publication (3), luck plays a role in research opportunities and experiences (2), students not being forthcoming about research in order to hoard research opportunities (1), confusion about research process (1), hard to balance schoolwork with research (1), issues with IRB approval (1), finding research in specialty of choice was easy (1), West Reading campus partnership with Reading Hospital has been good for mentorship/research (1), TriNetX is a plus (1)

- i think the hardest obstacle for drexel students applying to surgical subspecialities is not having specific mentors in the field to help guide/teach you how much research is required to be competitive to match to these specialities. neurosurgery i think has very little mentorship opportunities at drexel, and coming into my interest in the field (M3) so late in the game, i feel i havent had a lot of guidance from drexel on how i can make myself a strong applicant, but moreso from other students applying who have spoken to many other faculty members at different institutions.
- Required a lot of self directed cold emailing and hard work but was able to find success from being committed to the opportunities given to get future opportunities. Most opportunities I found were from my own cold emailing and not from DUCOM
- it has been especially hard to find research at Drexel for ophthalmology because there isn't a ton of info on webcampus about it, or previous students have done research elsewhere in there hometown etc
- It was pretty much impossible to find research in Uro and ENT through Drexel and the opportunities they shared. I felt like I had zero guidance in the process, and still struggle to utilize the career advising office because they have such limited availability (I have emailed multiple faculty members and they're always booked 1-2 months out)
- Drexel lacks opportunities to do clinical research. It is based on providers opening up their information to students to conduct chart reviews, but there is not always enough research to go around and the infrastructure around research, such as statisticians, funding to present your research, and research librarians to assist with specialty specific research is lacking and not sustainable. In order to do well you need to have a research background coming in or get invested in a research group early on, which is not feasible for a lot of students.
- Hard to find clinical research through drexel portal/faculty, its difficult to cold email if you do not know what specialty you want to do or have connections
- I found that many zoos could not take me because I did not go to their school and often DUCOM associated faculty did not respond to my cold emails about research.
- Pretty hard to find research, especially in interventional radiology. I had to really go out of my way to find things.
- I think I was lucky that our current ENT pathway director has a research fellow with a lot of projects. I was easily able to learn about these projects through an interest group meeting and was guided through the process of how to join. However, I have been looking into finding other research opportunities and it has been difficult to get ahold of faculty at other institutions.
- It has been very difficult to get projects, the Drexel ENT practice is poorly organized and the Penn and Jefferson organizations are hard to tap in to
- All of my research was with local Philly institutions outside of research, which I pursued intentionally as I want to match at those hospitals and think those connections will take my further
- DUCOM provides opportunities by being near other residency programs.
- The reason I ranked myself as a 5 is because I completed a research year that was very productive. I think without the research year I would have ranked it as 4 because I got involved with research early and pushed myself M1-3 because I knew research was important for ENT
- I got really lucky and reached out to the right person that was able to let me do a research year. However, this was an unpaid position and I had to do a Master's to get enough money to live. A research year is not usually a soft requirement for urology but it is the reason I had the opportunity to interview at top residency programs and expect to match really well. The year was as much about finding mentors and advocates as it was about the research.
- Took a research year for ophthalmology. Had an extremely difficult time finding opportunities.
- For ophthalmology: I did a competitive summer program after M1, and am now working with a Hopkins faculty remotely during M2 year. Other: I've kept up with the lab that I worked with in college/during my gap year and have gotten a few publications from this during med school. This research isn't related to my primary speciality, but I figured it was a good idea to keep up with it since it is fruitful with publications. That being said, I wish it was easier to find reliable publications in Ophthalmology, because I think that would add the most value to my application.
- I feel like the biggest challenge for finding research in Ophtho was not having connections/having a PI/mentor who was willing to take on students, especially since I only had bench research experience and not clinical research experience. The only person that ended up responding was a PI for bench research, which was not my preferred type of research to be doing but ended up doing it because I had no other options. The experience was good for gaining a mentor, but since bench research takes a long time, I likely won’t see any publications from it before applying.
- Compared to my previous research experiences, this lab was less prolific and it seemed underfunded. The laboratory wasn’t as up to par as other I have seen, with older reagents and at times somewhat questionable techniques. My project never really gained any ground and it’s very unlikely I’ll get any sort of publications from the experience. But the mentor was extremely kind and supportive, so there’s that!
- Rothman research is very hit or miss. Some people say they’re so involved meanwhile I did some work on one project, the project got dropped and then I kept attending the meetings and emailing for updates and have not heard from anybody
- It feels like it mostly comes down to luck and connections.
- I feel like this is a side hustle that none of the students really talk about but everyone is trying to get some. Like were all competing becuse theres 200 of us and since we dont have an affiliated hospital there arent a lot of spots. So everyone is not being very vocal about what they're doing
- very confusing at first, and I feel that the faculty-led zoom sessions on how to find research should be presented earlier in the M1 fall semester, as I feel I don't have a lot of time to find research and apply to MSSRF
- Most of my medical school research has been through Rothman doing data collection for the Spine Fellows, so I haven't really led my own study and will be lower on authorship lists. I did start a TriNetX project with Dr. Ilyas which morphed into 2 projects now. Overall, I think research has been challenging just to balance with schoolwork on top of finding opportunities or reaching out and finding mentors interested in supporting students with projects. I think that's mostly my fault though by not being proactive last year and cold-emailing PIs to pursue a summer opportunity for M1 summer.
- My research project fell through because we were unable to get the IRB approval for a second round of data collectionr. Without the approval we didn’t have enough data.
- It was not too challenging to find a research position and projects to work on. Working on exactly the research I'm interested was more difficult, but finding research in my specialty was fairly easy as a DUCOM student.
- Luckily as a West Reading student, we have a hospital here that has research opportunities and shadowing availability. Our pathway director also works here and our home rotation site for the pathway is with her, so the ophthalmology experiences I’ve had and the research I’ve been given has all been local with no formal vetting process. It’s in person research as well, so being here has been crucial.
- TriNetX seems really helpful for remote research.

(2) **(Optional) Describe what type of support DUCOM can provide students interested in research to improve their experience.**

Summary: More alumni connections in specific specialties (5), more specialty-specific mentors (5), more upperclassmen support (2), more resources for surgical subspecialties (2), email about research opportunities (2), list of research of opportunities (2), collaborations with other schools/institutions for more research opportunities (2), more TriNetX research mentors (2), case reports (1), more funding for conferences (1), statistical support (1), update list of research opportunities more frequently (1), more clinical research faculty in Philly area (1), more research funding (1), earlier exposure about how important research is for residency applications (1), formal research program as part of curriculum (1)

- Connecting more alumni/faculty in these competitive surgical sub specialities that have active research projects that students can get involved in.
- ENT applicants are lucky because we have Dr Sataloff as our Department chair and he is a very productive researcher. I think it could be beneficial to have DUCOM try to set up more connections with alumni to strengthen research opportunities
- Better alumni connections, resources for research at our regional campuses, not just in Philly
- I think DUCOM does a good job so far, but I think we all mostly rely on Dr. Ilyas to run our own TriNetX study's. I think if Drexel was able to expand their faculty to include more orthopedic surgeons who are interested in pursuing research would assist, as more "home" surgeons could be more committed to helping our students. I also think just connecting with DUCOM alumni who are now ortho attendings who do research would be beneficial as well.
- Connect us with Alumni for each specialties
- Research mentors and projects in place for specialties without a home program. A very simple, basic walkthrough of what kind of projects can be done with trinetx and how to use it- I attended all the training seminars but still was not able to understand until meeting with a fellow student who’d used it before.
- i think drexel can have a direct pathway for people interested in surgical subspecialities at M1, even if they change their minds at least they'll set themselves up to be competitive enough for whatever they end up applying into. this should include direct mentorship with a faculty that has real presence at drexel. we have some great mentors at AHN for neurosurgery, but thats another aspect that drexel students have to navigate themselves. i also had to do a lot of self-directed research to find a research year position in neurosurgery, and even then i feel as though i wasn't equipped to understand the impact of choosing a mentor who would help me be productive with my year and support me in my application and interests. mentorship is a huge deal in these smaller subspecialities, and coming from drexel its even more important to have some sort of connection that can stick their neck out for you to help you network to hopefully match. the mentorship is the main piece that drexel is missing.
- For urology specifically, it is difficult to work with our current pathway director. I don't know what could be done about that to be perfectly honest, but Urology is such a small field where you need to know people who know people to gain access to certain things..
- I think getting connected to the pathway director immediately is the main thing thats helped me get research. Because they are mentors that can really get you ready for residency applications, telling you what to do and what to avoid. I personally think that asking other students is ineffective. Second year students do have a little more experience than us but they are still figuring things out. I would much rather take advice from people that are already in residency or have experience with residency applications or know exactly how the later years of a career in medicine work.
- lists of labs looking for students to join from older students, older students pulling younger students into projects
- Lists of upperclassmen’s research experience and contact info if they are open to sharing
- Please provide more opportunities for other surgical specialties. There seems to be endless neurosurgery, urology, orthopedic, but not enough for general surgery, cardiothoracic, etc.
- Email us about research opportunities available
- more info sent out via email regarding open research opportunities to lessen burden on students in looking for research projects
- Mentors with specific projects listed! Had to cold email PIs and got very little response.
- Potentially a guide made for each specialty that is regularly updated containing institutions and/or faculty that are looking for medical students to assist with research may help.
- I think if they had more collaborations with other schools especially in specialties where we don’t have a home program, it would allow us to network better and get more opportunities that way.
- Relationships with other health facilities in the city, the singular option within the DUCOM community is not sufficient to accomodate all interested students
- More trinetx projects in the database, more virtual opportunities for clinical research given how majority of students don’t get access to in person work at our rotation sites and the Tower Health network more local to west reading students isn’t all encompassing either
- Our pathway directors are fantastic! They are super knowledgable and a great resource. The one thing I wish I had more access to is case reports, so maybe some sort of system where we could get matched with doctors in our field and given case reports would be nice. Even just 2-3 a year would make a big difference.
- Increase funding for attending conferences both near and far so that attending multiple conferences a semester or year is not draining the loan money.
- Statisticians- generally available to work on student projects, more faculty who will allow students to be involved in research
- Ducom has a list online, but most of those opportunities have already been filled. The list should be updated more frequently
- build out clinical faculty in the Philly area. That is a huge detriment to student education and research opportunities
- DUCOM just needs to increase their research funding in my opinion. If we can’t obtain more R1 grants then maybe the university or a generous donor can help supplement some new, exciting projects that will attract qualified staff. If Queen Lane is our research facility then that’s an issue, too. Queen Lane is too far and the building/labs are old and pretty desolate, sorry! It’s not an attractive lure for medical students, so it doesn’t surprise me that students look elsewhere to get research experience.
- I wish I knew earlier how important it is to have published work. I never planned on a competitive specialty and thought that only competitive specialties needed it - but that does not feel accurate anymore!
- A formal research program would be nice. For example, some of my friend’s medical school requires that they do a certain amount of research and have many options to connect them with PIs.
